# Sex differences in the relationship between depression and Alzheimer’s disease—mechanisms, genetics, and therapeutic opportunities

**DOI:** 10.3389/fnagi.2024.1301854

**Published:** 2024-06-05

**Authors:** Yu-Han Chen, Zhi-Bo Wang, Xi-Peng Liu, Jun-Peng Xu, Zhi-Qi Mao

**Affiliations:** ^1^The First Clinical Medical School, Hebei North University, Zhangjiakou, China; ^2^Innovation Center for Neurological Disorders and Department of Neurology, Xuanwu Hospital, Capital Medical University, National Center for Neurological Disorders, Beijing, China; ^3^Department of Neurosurgery, The First Affiliated Hospital of Hebei North, Zhangjiakou, China; ^4^Department of Neurosurgery, The First Medical Center of Chinese PLA General Hospital, Beijing, China

**Keywords:** Alzheimer’s disease, depression, sex differences, mechanisms, genetics, therapeutic opportunities

## Abstract

Depression and Alzheimer’s disease (AD) are prevalent neuropsychiatric disorders with intriguing epidemiological overlaps. Their interrelation has recently garnered widespread attention. Empirical evidence indicates that depressive disorders significantly contribute to AD risk, and approximately a quarter of AD patients have comorbid major depressive disorder, which underscores the bidirectional link between AD and depression. A growing body of evidence substantiates pervasive sex differences in both AD and depression: both conditions exhibit a higher incidence among women than among men. However, the available literature on this topic is somewhat fragmented, with no comprehensive review that delineates sex disparities in the depression–AD correlation. In this review, we bridge these gaps by summarizing recent progress in understanding sex-based differences in mechanisms, genetics, and therapeutic prospects for depression and AD. Additionally, we outline key challenges in the field, holding potential for improving treatment precision and efficacy tailored to male and female patients’ distinct needs.

## 1 Introduction

Alzheimer’s disease (AD) is a leading neurodegenerative disorder, characterized by cognitive deficits, behavioral changes, and memory loss (Nebel et al., 2018; Depp et al., 2023). Recognized as a multifactorial disorder with diverse etiologies (Guo et al., 2022; Uras et al., 2023), emerging evidence highlights sex differences as a significant contributor to AD’s heterogeneity, influencing prevalence, disease progression, risk factors, and outcomes (Tremblay et al., 2023). Women, who represent about two-thirds of AD patients, exhibit a faster cognitive decline and higher susceptibility to AD (Hampel et al., 2018; Levine et al., 2021; Martinkova et al., 2021; Cui et al., 2023). This increased vulnerability is partly due to women’s longer average lifespan compared to men’s (Hampel et al., 2018; Guo et al., 2022; Kommaddi et al., 2023). For instance, women outlive men by approximately 4.5 years globally, with a higher number of women reaching the age of 85 and beyond (Zhu et al., 2021). This longevity contributes to their amplified risk of developing AD with age. However, age alone does not fully explain the higher incidence and lifetime risk of AD in women (Cui et al., 2023; Kommaddi et al., 2023). Therefore, exploring the genetics and mechanisms underlying sex differences in AD is crucial for developing sex-specific therapeutic strategies.

While AD predominantly impacts memory, nearly all patients exhibit psychological symptoms, including depression, which worsens with AD progression (Lyketsos et al., 2011; Santos et al., 2016). Depression is a prevalent and debilitating condition that impairs quality of life and imposes a significant socio-economic burden (Labonté et al., 2017; Malhi and Mann, 2018). Although it affects both sexes, women are twice as likely to suffer from depression, often experiencing more severe symptoms and functional impairments (Labonté et al., 2017; Kundakovic and Rocks, 2022). Depressed individuals are over twice as likely to develop AD dementia compared to those without depression (Elser et al., 2023), underlining a notable link between the two conditions. Interestingly, both depression and AD are more common in women (Wang et al., 2022a), yet studies suggest that men with depression might be at a higher risk of developing AD, and they are more likely to show depressive symptoms following AD onset (Fuhrer et al., 2003; Dal Forno et al., 2005; Underwood et al., 2019; Tremblay et al., 2023).

In this review, we describe key progress in investigating sex differences in the mechanisms, genetics, and therapeutic approaches for depression and AD so far, along with current studies aimed at improving treatment precision and efficacy by identifying sex-specific traits of depression and AD, expediting their fate acquisition in precision medicine. We anticipate the course of this evolving line of research, which increasingly emphasizes sex variations in etiology and genetics. This enhanced focus holds promise for the eventual development of more efficacious and broadly relevant treatments for AD and depression in the future.

## 2 Sex differences in Alzheimer’s disease

### 2.1 Alzheimer’s disease

AD stands as the primary cause of dementia, rapidly becoming one of the most financially demanding, fatal, and burdensome conditions today and presents as a clinical syndrome characterized by progressive cognitive impairment that affects multiple domains or by neurobehavioral symptoms of sufficient severity to distinctly impact daily functioning (Scheltens et al., 2021). Globally, AD affects approximately 50 million individuals, with its prevalence projected to increase by about 70% by the 2050s (Cui et al., 2023; Fronza et al., 2023). Most cases arise after age 65, termed late-onset AD, whereas cases before age 65 are less common, constituting about 5.5% and referred to as early-onset AD (Long and Holtzman, 2019; Seath et al., 2023). Furthermore, AD is biologically defined by specific neuropathological features (Jack et al., 2018), including extracellular accumulation of β-amyloid (Aβ) in diffuse and neuritic plaque forms alongside the existence of intraneuronal neuropil threads and neurofibrillary tangles within dystrophic neurites consisting of polymerized hyperphosphorylated tau protein (Long and Holtzman, 2019). The amyloid cascade hypothesis proposed in 1992 by Hardy and Higgins suggests Aβ deposition in the brain initiates AD pathogenesis (Hardy and Higgins, 1992), with Aβ accumulating in cortical extracellular plaques approximately 10–30 years before dementia onset, contributing to subsequent the deposition of tau, synaptic loss, and cognitive decline (Long and Holtzman, 2019; Wang et al., 2022b). However, the hyperphosphorylated and aggregated tau protein may be the primary driving factor – and potentially the sole determinant – of neurodegeneration in AD (Aschenbrenner et al., 2018; Long and Holtzman, 2019; Wang et al., 2022b). These complexities contribute, at least in part, to the challenges in clinical intervention and diagnostics of patients with AD.

To date, four conventional Food and Drug Administration (FDA)-approved drugs are available for treating cognitive performance and daily functioning in AD dementia: cholinesterase inhibitors (ChEIs) and anti-N-methyl-D-aspartate receptor modulators. However, these drugs confer benefits only during the initial year of treatment and have demonstrated limited long-term efficacy (Wang et al., 2022b). In 2021, the FDA granted accelerated approval for aducanumab, an anti-Aβ drug designed for AD. However, this decision spurred intense debates over the insufficiency of evidence regarding its efficacy (Jaffe, 2021; Zhang Y. et al., 2023). More recently, lecanemab, another anti-Aβ drug, received full FDA approval (Mahase, 2023; Zhang Y. et al., 2023), with emerging evidence indicating a 27% reduction in cognitive decline rate over 18 months in treated AD patients (Høilund-Carlsen et al., 2023). Despite these drugs’ proven beneficial effects in AD clinical trials, the risk of side effects, such as amyloid-related imaging abnormalities (ARIA), including hemosiderosis (ARIA-H), cerebral edema (ARIA-E), and cerebral microhemorrhage, should be acknowledged (Jeremic et al., 2023). Furthermore, as disease mechanisms become better defined, disease-modifying treatments such as anti-inflammatory agents, genetic modifications, and hormone therapies can target various factors to enhance cognitive function and halt disease progression (Long and Holtzman, 2019; Vegeto et al., 2020; Wang et al., 2022b).

### 2.2 The role of sex differences in AD

**Figure 1:**
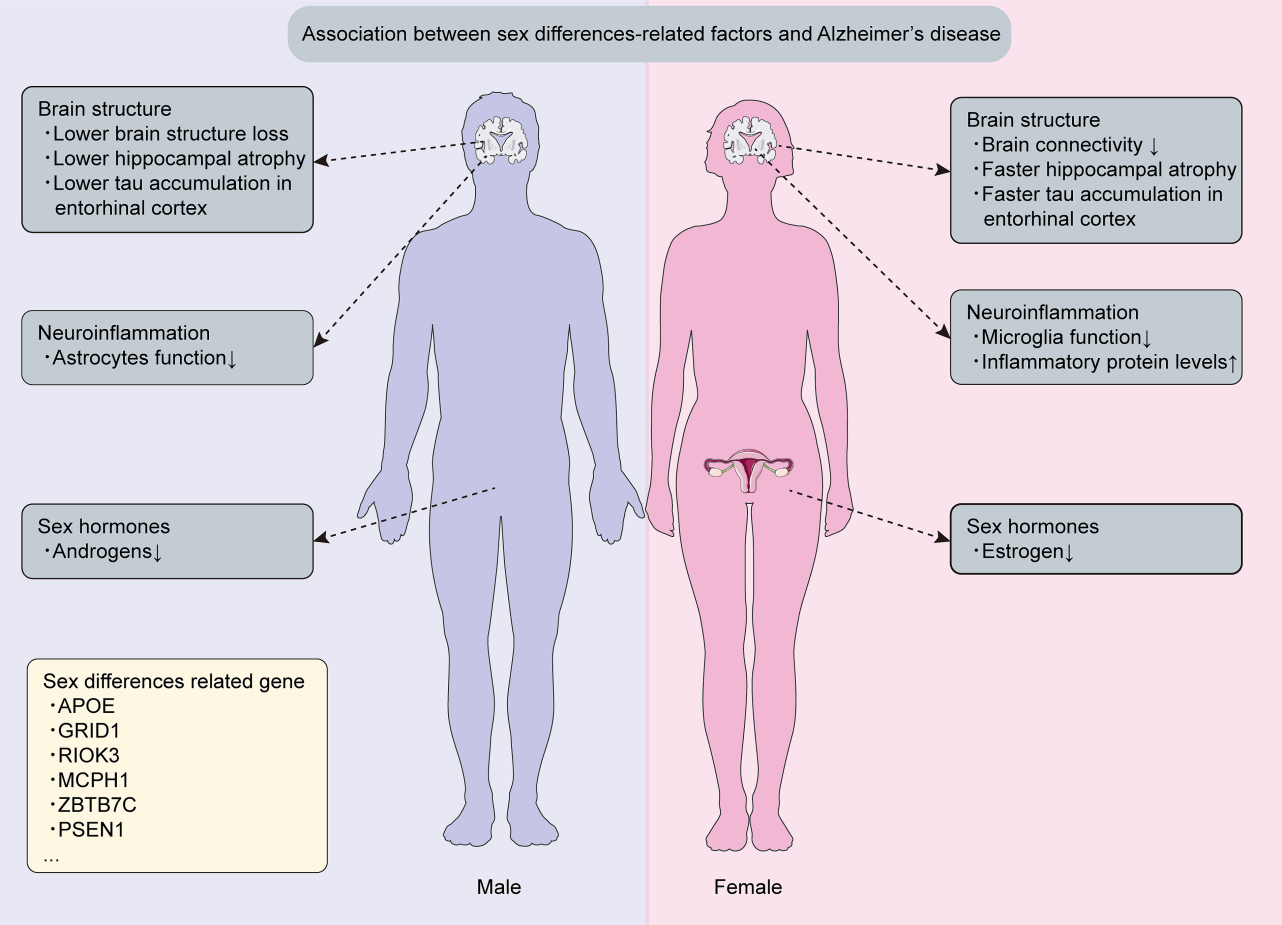
Sex differences related mechanisms and genetics in Alzheimer’s disease. This figure is about key discoveries regarding sex differences related mechanisms and genetics associated with Alzheimer’s disease.

Nearly two-thirds of AD patients are female, experiencing more pronounced cognitive impairment than males at an equivalent disease stage (Levine et al., 2021; Zhu et al., 2021; Lansdell et al., 2023; Lutshumba et al., 2023). Although sex differences in AD dementia are evident, the underlying reasons remain unclear. Closing this knowledge gap is crucial, as comprehending mechanisms and genetics driving distinct susceptibility between AD-affected males and females informs personalized prevention and treatment strategies for this pervasive neurodegenerative concern (Lutshumba et al., 2023). Therefore, this section provides current evidence on sex differences in AD, encompassing mechanisms, genetics, and therapeutic responses (Figure 1).

#### 2.2.1 Sex differences in AD’s mechanisms and genetics

##### 2.2.1.1 Sex hormones

Sex hormones, particularly estrogens and androgens, were found to significantly influence the sexual dimorphism observed in AD (Sochocka et al., 2023; Yeung et al., 2023). Another study also reported the role of sex hormones in creating distinct sex-based disparities in AD pathogenesis (Pike, 2017). These findings underline the significance of scrutinizing sex hormones in future AD research on sex differences.

###### 2.2.1.1.1 Estrogen

Estrogens exert extensive neuroprotective effects in the adult brain, bolstering neural resilience and function while also specifically attenuating multiple aspects of AD-related neuropathology (Pike, 2017).

Additionally, estrogens play a vital role in the sexual differences observed in AD. Estrogen levels in women markedly decrease during menopause, and research by Karamitrou indicates that an earlier onset of menopause is associated with an increased risk of dementia (Uddin et al., 2020; Karamitrou et al., 2023; Mills et al., 2023). Menopausal reduction in β-estradiol can trigger S-nitrosylated C3 formation, resulting in synaptic phagocytosis, synapse loss, and ensuing cognitive decline in women with AD (Yang et al., 2022). Studies have demonstrated that estrogens protect against cognitive impairment of AD resulting from a cholinergic deficit in women experiencing premature menopause in middle age (de Torre et al., 2022; Sochocka et al., 2023). In addition, events that reduce lifetime exposure to estrogens are generally associated with an increased risk of AD among women. However, among men, estrogens do not show age-related reduction and are not significantly associated with the risk of AD (Pike, 2017).

###### 2.2.1.1.2 Androgens

Testosterone, an androgen, acts as a key sex-related factor with a neuroprotective role by improving synaptic signaling, reducing Aβ deposition, and diminishing neuronal death (Bianchi, 2022; Kusters et al., 2023). Recent findings indicate minimal or insignificant effects of androgens in women, whereas higher androgen levels correlate with reduced AD risk in men (Kusters et al., 2023). Notably, aging men exhibit increased estrogen levels due to androgen aromatization, unlike women, providing enhanced cognitive protection in AD (Sumien et al., 2021). Overall, these studies offer fresh insights into men’s lower susceptibility to AD compared with those of women.

##### 2.2.1.2 Brain structure

Previous experiments have shown marked differences between male and female brains (Zhu et al., 2021), and therefore, delving deeper into the uneven impacts of sex on alterations in brain structures could furnish insights into the fundamental rationales and mechanisms underpinning the disparities in male and female brains while progressing through AD development. Men generally have larger total brain volumes and head sizes, which suggests a higher cerebral reserve capacity – the brain’s resilience to neuropathological damage – compared to women. This greater reserve may enable men to withstand more extensive pathological insults (Riley et al., 2002; Giedd et al., 2012; van Eijk et al., 2021). In addition, men with AD tend to experience less brain atrophy and subsequent memory loss compared to women with AD (Sandau et al., 2022). The connectivity between the hippocampus, brainstem, and precuneus cortex shows more strength in men than in women (Williamson et al., 2022), intensifying female susceptibility to swift hippocampal atrophy and potential cognitive regression. Notably, the accumulation of phosphorylated tau in the entorhinal cortex occurs more rapidly and to a greater extent in women than in men, contributing to the elevated susceptibility of women to AD compared with that of men (Hu et al., 2021; Chen X. L. et al., 2023), which indicates that the mechanisms that contribute to the entorhinal cortex’s notable susceptibility to AD could be closely connected to those responsible for the sex differences in AD pathogenesis.

##### 2.2.1.3 Neuroinflammation

Neuroinflammation is widely acknowledged as a pivotal element in AD’s pathogenesis, contributing to neurodegeneration and cognitive impairment. It is predominantly attributed to central nervous system (CNS) immune cell dysfunction, particularly involving astrocytes and microglia. A large number of studies have explored the impact of sex on neuroinflammation in AD (Guillot-Sestier et al., 2021; Singh, 2022; Cui et al., 2023; Tamburini et al., 2023). Specifically, one study that explored sex differences in microglia using post-mortem tissue from male and female AD patients revealed a noteworthy increase in the count of dystrophic microglia in AD females (O'Neill et al., 2022). Another animal study concluded that gene upregulation linked to microglial activation was more prominent in female APP/PS1 mice compared with male mice and this difference in gene expression contributed to decreased microglial phagocytic activity and heightened amyloidosis in females (Guillot-Sestier et al., 2021). Moreover, the study revealed that sex-related variations observed in animal models were also evident in human AD patient post-mortem brain tissues. Specifically, in the post-mortem brain tissue of female AD patients, there was a significant increase in amyloid plaque coverage in the cortical regions compared to male patients. In contrast, male patients showed more pronounced amyloid staining in their vasculature. Moreover, microglia from male AD patients predominantly exhibited an amoeboid shape with little morphological heterogeneity. In contrast, brain tissues from female AD patients demonstrated marked heterogeneity, with few amoeboid cells, some ramified cells, and numerous rod-shaped microglia (Guillot-Sestier et al., 2021). Furthermore, research demonstrated that female mice astrocytes displayed a more robust inflammatory response marked by elevated IL-6, IL-1β, and NF-κBIA levels, while male mice astrocytes showcased elevated anti-inflammatory cytokine IL-10 levels and increased survival (Lennol et al., 2021). Even though the majority of studies suggest amplified neuroinflammation in females, one study that employed Tau P301S transgenic mice unveiled more significant alterations in inflammatory induction and astrocyte activation in male mice than in female mice (Sun et al., 2020). In conclusion, these findings collectively confirm intensified microglial activation and increased inflammation in females with AD. However, the results of prior investigations pertaining to sex differences in astrocyte activation exhibit inconsistency. Specifically, male astrocyte activation has been concluded to display a greater increase compared with female astrocyte activation.

##### 2.2.1.4 Sex differences in AD’ s genetics

###### 2.2.1.4.1 Sex-specific genes in AD

Sex disparities significantly influence AD susceptibility, yet their distinct roles have been underexplored in AD genetics (Nebel et al., 2018). Therefore, it is essential to identify the genetic factors that underlie sex differences in AD, as this can lead to personalized treatment and enhance the accuracy of disease risk assessment. The X chromosome constitutes 5% of the genome in both men and women (Davis et al., 2021). A transcriptome analysis conducted on the prefrontal cortex of AD patients of both sexes unveiled that the gene expression of the X chromosome (29 genes) was significantly linked to cognitive alterations exclusively in women, whereas this association was not observed in men, wherein 19 genes of the X chromosome exhibited an association with decelerated cognitive decline among women (Davis et al., 2021). Furthermore, one study identified *ZBTB7C* as a new sex-specific AD risk factor and through meticulous scrutiny found that the minor allele *rs1944572* of *ZBTB7C* raised AD risk in women but safeguarded men (Prokopenko et al., 2020). Notably, presenilin-1 (*PSEN1*) mutations cause most familial AD cases (Hurley et al., 2023), and emerging findings indicate that female *PSEN1* carriers exhibit a higher level of plasma neurofilament light (NFL), a biomarker representing neurodegeneration, compared with male carriers (Vila-Castelar et al., 2023), suggesting that women may experience a higher rate of neurodegeneration than men.

###### 2.2.1.4.2 *ApoE* gene

The *apolipoprotein E* (*ApoE*) gene is the dominant and strongest genetic risk factor for AD, displaying sex differences in its association with the disease (Hohman et al., 2018; Zhang L. et al., 2023). More particularly, AD is more frequently developed in female carriers of *ApoE-ε4* compared with age-matched males (Sandau et al., 2022). The *ApoE* gene, located on chromosome 19, consists of three common alleles that encode three protein isoforms: *ApoE2, ApoE3,* and *ApoE4*, which differ at two amino acid positions (Raulin et al., 2022; Zhang L. et al., 2023). Notably, among these isoforms, *ApoE4* significantly increases the risk of AD, while *ApoE2* reduces the risk by approximately 50% and contributes to longevity (Raulin et al., 2022). The interactive effects of sex and *ApoE* genotype impact wide neuropathological processes linked to AD. During the cognitively normal stage of AD, *ApoE-ε4* noncarriers of female display significantly higher Aβ levels compared with males, whereas *ApoE-ε4* carriers among males exhibit considerably greater Aβ burden than noncarriers (Pan et al., 2023). Furthermore, mild cognitive impairment (MCI) represents the prodromal stage of AD (Reas et al., 2023), and previous studies indicated that male *ApoE-ε4* carriers with MCI exhibited higher levels of amyloid deposition in the older age group, while female *ApoE-ε4* carriers with MCI showed increased amyloid deposition in the younger age group (Wang J. et al., 2023). The impact of *ApoE* on the pathological processes of AD in various brain regions also differs between sexes. More specifically, a quantitative 18F-flortaucipir PET study conducted on individuals with cognitive impairment revealed that the dosage of the *ApoE-ε4* gene had a sex-specific effect on tau deposition in regions like the amygdala, medial temporal lobe, lateral temporal lobe, posterior cingulate cortex, entorhinal cortex, parahippocampal gyrus, and inferior temporal regions (Yan et al., 2021). Interestingly, this *ApoE-ε4* allele’s dose-dependent effect on specific tau deposition in brain regions was observed only in men, not women (Yan et al., 2021).

##### 2.2.1.5 Sex differences in AD’s therapeutic opportunities

The heterogeneity in the progression and manifestation of disease among AD patients has been recognized as a pivotal issue in the present approach for developing AD novel therapies, and sex is a critical variable contributing to the heterogeneity of this disease (Ferretti et al., 2018), making it essential to focus on considering the role of sex differences in AD therapies. The current treatment strategies regarding sex differences in AD are showed in Table 1.

**Table 1:** Sex differences in Alzheimer’s disease therapeutic opportunities.

Drugs		Therapy effects	
		Male	Female
ChEIs	Rivastigmine	++^a^	++^b^
	Donepezil	NA	NA
	Galantamine	NA	NA
Anti-NMDA	memantine	NA	−
Disease modifying therapy	Lecanemab	++	+
	Donanemab	+	++
HT	Estrogen	NA	++
	Androgen	++	+

^a^represents that the therapeutic efficacy occurred in the early stage of AD; ^b^represents that the therapeutic efficacy occurred in the later stage of AD; + represents the general therapeutic efficacy; ++ represents the better therapeutic efficacy; − represents poor therapeutic efficacy; NA represents that the therapeutic efficacy is unclear.

AD, Alzheimer’s disease; ChEIs, Cholinesterase inhibitors; Anti-NMDA, anti-N-methyl-D-aspartate; HT, Hormone therapy.

###### 2.2.1.5.1 Cholinesterase inhibitors and anti-NMDA

The FDA has sanctioned three ChEIs: rivastigmine, donepezil, and galantamine, along with the uncompetitive NMDA receptor antagonist called memantine, for mitigating dysfunction and cognitive impairment associated with symptomatic AD (Long and Holtzman, 2019; Thakral et al., 2023). However, existing pharmacological interventions for AD dementia provide merely temporary relief from symptoms instead of modifying the underlying disease progression (Marasco, 2020; Zhang Y. et al., 2023). Noteworthy is a study indicating a higher likelihood of early-onset AD in males treated with memantine, whereas females were more linked to late-onset AD (Miller et al., 2022), which this divergence in association could possibly stem from sex-related differences in the effectiveness of memantine treatment. However, understanding the precise mechanism requires further investigation and worth noting is memantine’s potential slightly negative impact on female rat skeletal health relative to males (Londzin et al., 2023). Interestingly, during the prodromal stages of AD, treatment with rivastigmine has been observed to delay the progression from MCI to AD in women, but not in men. In contrast, the treatment of ChEIs in the advanced stages of AD has exhibited more selective and pronounced beneficial effects in men (DuMont et al., 2023). Despite the limited attention given to the existence of sex differences regarding the efficacy of memantine and ChEIs in AD thus far (Canevelli et al., 2017), this topic represents a significant area that warrants future research in AD.

###### 2.2.1.5.2 Anti-amyloid monoclonal antibody drugs

The amyloid cascade hypothesis is central to AD pathogenesis and provides a valuable conceptual framework for therapeutic advancements in this domain (Long and Holtzman, 2019; Yadollahikhales and Rojas, 2023). This hypothesis focuses AD treatment approaches on amyloid clearance to mitigate disease progression. Thus far, both lecanemab and aducanumab, targeting Aβ drugs in AD, have achieved key milestones with FDA approval through an accelerated approval pathway (Yadollahikhales and Rojas, 2023). However, one recent investigation has indicated that donanemab, another anti-Aβ drug for AD, likewise showcases clinical effectiveness (Høilund-Carlsen et al., 2023). Crucially, sex-based differences in responses to lecanemab and donanemab in AD patients might exist. One study’s primary endpoint was the alteration in the Clinical Dementia Rating–Sum of Boxes score, a scale ranging from 0 to 18, where higher values signify greater impairment. The findings of this study indicated that for male participants, the between-group comparison (lecanemab vs. placebo) exhibited a statistically significant difference, and in contrast, the same comparison among female participants lacked statistical significance, which suggested that lecanemab’s efficacy might be limited to men only (van Dyck et al., 2023). On the other hand, another study involved a subgroup analysis, assessing the change in the integrated Alzheimer Disease Rating Scale (iADRS) score, which ranges from 0 to 144, with lower scores indicating greater impairment, and interestingly, this analysis revealed the existence of sex differences in the efficacy of donanemab, showing that it may be effective exclusively in women (Sims et al., 2023). Further exploration of the causes and mechanisms underlying the observed sex differences between lecanemab and donanemab is essential in future research.

###### 2.2.1.5.3 Hormone therapy (HT)

Though hormone therapy (HT) holds potential for AD treatment, evident sex differences in treatment response exist. Overall, women appear to benefit more from HT in AD than men do, often linked to the more pronounced estrogen decline following menopause (Karamitrou et al., 2023). A prior study corroborates the idea that females may be protected by HT compared with men, possibly delaying AD onset as long as estrogen levels remain stable (Kommaddi et al., 2023).

Significantly, female sex hormones exhibit anti-aging properties and harbor enduring neuroprotective effects (Sochocka et al., 2023). Consequently, employing menopausal HT from the onset of menopause until the age of 60 could potentially establish a “window of opportunity” for diminishing the risk of MCI and AD among women in their later years (Sochocka et al., 2023). However, it is essential to acknowledge that recent findings from one study concerning HT in AD treatment have yielded mixed results, implying an elevated risk of AD in women undergoing prolonged HT (Mills et al., 2023). Another emerging study also highlights a 24% higher AD dementia risk linked to estrogen–progestin treatment during perimenopause (Pourhadi et al., 2023).

The effectiveness of androgen therapy in preventing and treating AD in men with age-related testosterone decline is supported (Rosario et al., 2012), and recent evidence indicates that higher androgen concentrations correlate with reduced AD risk in men, with minimal effects in women (Kusters et al., 2023). In contrast, a randomized controlled trial reported that 1 year of testosterone treatment in men with AD did not show any improvement in memory or other cognitive functions compared with placebo (Resnick et al., 2017).

## 3 Sex differences in depression

### 3.1 Depression

Depression is a prevalent and debilitating disease that significantly reduces the quality of life and is associated with an increased risk of suicide. It frequently recurs, with the risk increasing after each episode. Approximately 80% of patients experience at least one further episode during their lifetime, contributing to a major global mental health and economic burden and making it the leading cause of mental-health-related disability worldwide (Malhi and Mann, 2018; Marwaha et al., 2023). The World Health Organization (WHO) ranked major depressive disorder (MDD) as the third leading cause of global disease burden in 2008 and projected it to become the primary cause by 2030 (Malhi and Mann, 2018). For many individuals with depression, onset occurs in mid-to-late adolescence (e.g., ages 14 to 25 years), with a median 12-month prevalence of 4–5% in this age group (Thapar et al., 2012). MDD also negatively affects relationships, employment, and education, and it is prospectively associated with obesity, early mortality, and cardiac disease (Marwaha et al., 2023). Furthermore, while existing antidepressant treatments prove effective, a substantial proportion of individuals with MDD (about one-third to half) do not have respond to multiple antidepressants, and more may only achieve a partial response (Cipriani et al., 2018). Additionally, depressed patients typically have to await at least 4 weeks before an underlying response to current antidepressants occurs, and these medications often come with numerous side effects, such as loss of libido, agitation, headache, anxiety, and gastrointestinal symptoms, among others (Marwaha et al., 2023). Therefore, it is crucial to develop new agents or treatment modalities that present a more rapid onset of action, better tolerability, and greater effectiveness than existing antidepressants for individuals who fail to respond to current treatments.

### 3.2 The role of sex differences in depression

**Figure 2:**
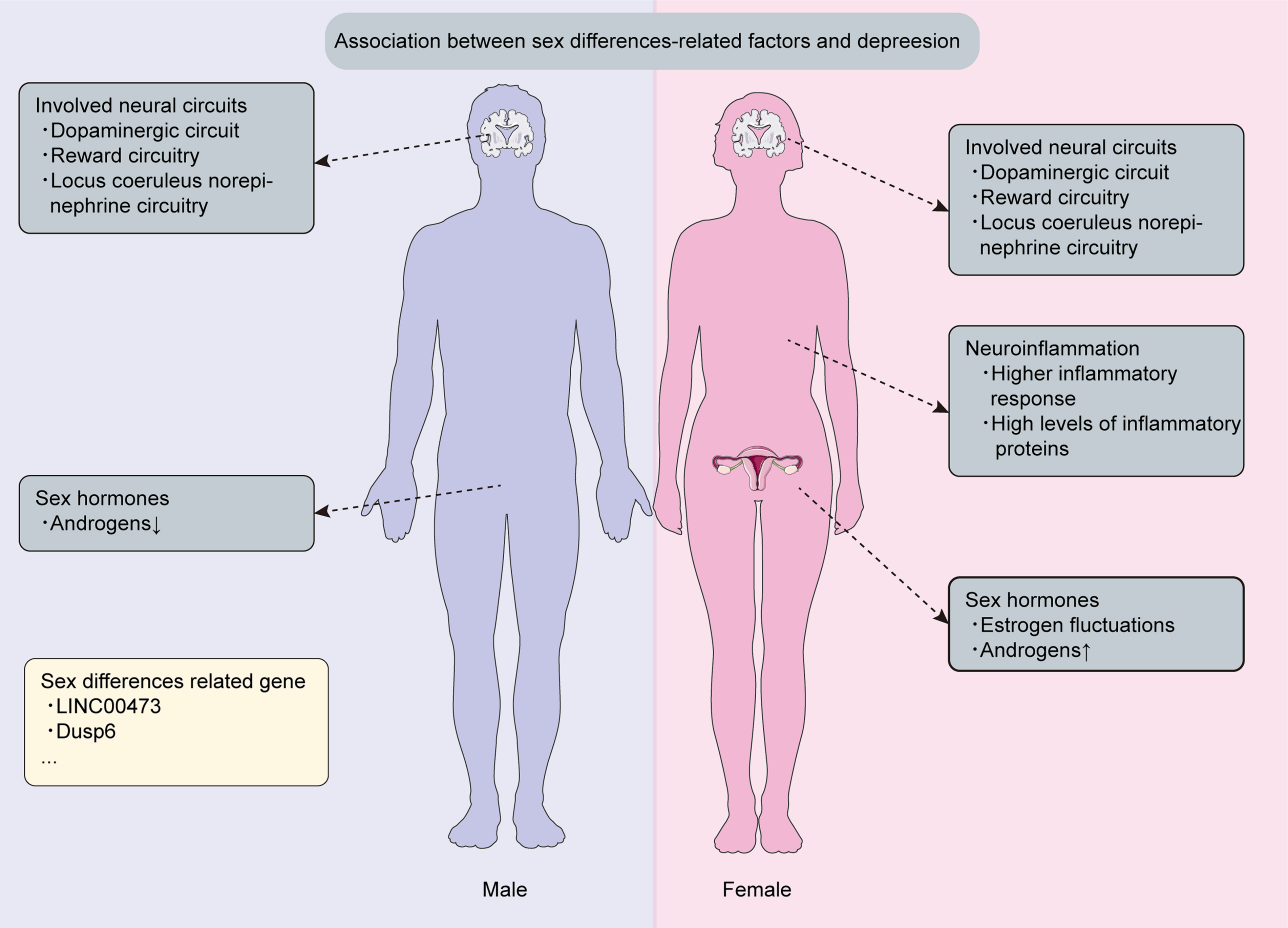
Sex differences related mechanism and genetics in depression. This figure is about key discoveries regarding sex differences related mechanisms and genetics associated with depression.

Depressive disorders are evenly distributed between boys and girls in childhood. However, a sex imbalance emerges at the age of 12 and peaks during adolescence, with young girls being up to three times more likely to be affected than young boys (Rossi et al., 2022). Furthermore, longitudinal studies conducted across diverse populations worldwide have confirmed that women are 2–5 times more likely to suffer from depression during perimenopause compared to the late premenopausal period (Bromberger and Epperson, 2018). Depression is more prevalent in women than in men (Kundakovic and Rocks, 2022), suggesting the existence of sex differences in depression, and importantly, conducting a thorough investigation of the genetic and mechanistic sex differences that contribute to depression is crucial for the development of more effective and universally satisfactory therapies (Figure 2).

#### 3.2.1 Sex differences in depression’s mechanisms and genetics

##### 3.2.1.1 Sex hormones

Females are twice as prone to depression than men are (Kundakovic and Rocks, 2022), displaying a stronger inclination toward severe symptoms (Labonté et al., 2017), and yet the precise mechanisms behind this remain unclear. Epidemiological investigations have documented that women manifest a heightened propensity for depressive disorders during different phases of hormonal fluctuation, such as puberty and menopause (Morssinkhof et al., 2020), suggesting that sex hormones might significantly contribute to this disparity. In this context, we delve into the discussion of three pivotal sex hormones: estrogen, androgens and ovarian progesterone.

###### 3.2.1.1.1 Estrogen

Research reveals that puberty-related hormonal changes contribute to sex-based disparities in depression prevalence (Morssinkhof et al., 2020). Notably, females who experience early onset of puberty face an elevated vulnerability to depression compared with their peers (MacSweeney et al., 2023), and women in the perimenopausal and postmenopausal stages exhibit a significant association with depressive disorders (Zhang J. et al., 2023), which may arise from the fact that depressive symptoms during these phases are more closely linked to fluctuations in estrogen levels. Significantly, administering exogenous estrogen can alleviate the severe hormonal fluctuations experienced by women during perimenopause and postmenopause. Intriguingly, the effectiveness of exogenous estrogen in mitigating the adverse effects of estrogen fluctuations depends more on the age of the women rather than the dosage of the administered estrogen (Zhang J. et al., 2023). Overall, a relative fluctuation in estrogen appears to correspond with heightened depressive symptoms (Morssinkhof et al., 2020), and these findings contribute to the partial elucidation of the sex differences in depression prevalence.

###### 3.2.1.1.2 Androgens

Androgens, including testosterone, dehydroepiandrosterone (DHEA), androstenedione, and 5α-dihydrotestosterone (5α-DHT), play a pivotal role in depression as sex-related factors (de Wit et al., 2021; Bianchi, 2022; Kische et al., 2022), and the association between depression and androgens has been well-established (Kische et al., 2022), with notable sex differences. Specifically, hypoandrogenic men exhibit a higher prevalence of MDD, while women with higher levels of free testosterone or supraphysiological androgens show a higher MDD prevalence (de Wit et al., 2021).

Notably, substantial studies suggests that low testosterone levels in aged men are correlated with an increased incidence of depression (Jankowska et al., 2010; Hauger et al., 2022; Handelsman and Wittert, 2024). Furthermore, one study demonstrated that DHEA and testosterone exhibited inverse associations with MDD among males, whereas no significant associations were observed among females (Kische et al., 2022).

###### 3.2.1.1.3 Ovarian progesterone

Women exhibit a higher tendency toward depression during menopause compared to the premenopausal phase, possibly due to the cessation of ovarian function and a decline in serum progesterone levels (Sovijit et al., 2021). However, the role of ovarian progesterone in depression remains controversial. One study suggests that reduced activity of progesterone receptors may contribute to depression-like behaviors in mice (Beckley et al., 2011). In addition, another study observes that progesterone treatment in ovariectomized (OVX) mice can ameliorate depressive and anxious behaviors by altering the gut microbiome composition, particularly by increasing the lactobacillus count (Sovijit et al., 2021). Yet, it’s important to note that research among rural Indian women found no significant link between progesterone and symptoms of depression (Walther et al., 2019). Similarly, a study on premenstrual dysphoric disorder (PMDD) among Taiwanese women reported no significant statistical correlation between progesterone levels and depression or anxiety scores (Hsiao et al., 2004).

##### 3.2.1.2 Neural circuits

Depression is frequently conceptualized as “circuitopathy” (Lu et al., 2022), displaying sex-specific variations. However, the intricate mechanisms that contribute to the sexual dimorphism in neural circuits associated with depression remains elusive. The prominent projection of the dopaminergic circuit connects the ventral tegmental area (VTA) in the midbrain to the basolateral amygdala (BLA) (Manion et al., 2022), which plays a significant role in emotional processing related to depression. One study has identified that although the size of BLA and the density of dopaminergic axons within BLA are similar between female and male mice, the density of dopaminergic synaptic boutons in the BLA is significantly higher in male brains compared with female brains (Manion et al., 2022), indicating that men may be more effective in alleviating their depressive symptoms than women are. In addition, anhedonia, mediated by the reward circuitry, including the nucleus accumbens (NAc), is a key feature in patients with MDD, which mainly manifests as a difficulty using rewards to modulate depressive behavior. The NAc receives dopaminergic inputs from the VTA, signaling motivational salience, and then outputs to basal ganglia circuits to drive motivated actions related to depressive diseases (Bangasser and Cuarenta, 2021). Interestingly, although studies in rodents have reported stress-induced sex differences in the NAc circuitry, functional changes in the NAc and anhedonia-related structural changes do not differ by sex in humans (Williams et al., 2020; Bangasser and Cuarenta, 2021). Furthermore, another study using animal models of depression has indicated sex differences in the locus coeruleus (LC) norepinephrine (NE) circuitry, and the LC NE circuitry plays a crucial role in mediating sex differences in arousal, which may contribute to several symptoms of MDD, such as lack of concentration, restlessness, and rumination, among others (Bangasser and Cuarenta, 2021; Williams et al., 2022). The dendritic architecture of LC neurons in female rats is more complex compared with that in males, and female LC dendrites receive significantly more synaptic input (Williams et al., 2022), which may underlie the heightened arousal in females, potentially contributing to the sex differences in the incidence of depressive symptoms.

##### 3.2.1.3 Neuroinflammation

Neuroinflammation is implicated in depression, significantly contributing to its pathophysiology (Sørensen et al., 2022, 2023). MDD patients exhibit elevated pro-inflammatory cytokine levels, and post-mortem examinations of depressed patients’ brains have found the presence of neuroinflammation (Santos et al., 2016; Malhi and Mann, 2018). However, growing evidence suggests sex-specific differences in underlying neuroinflammation mechanisms (Achtyes et al., 2020; Wang et al., 2022a). For instance, in one study, both male and female mice underwent a four-week intervention of chronic unpredictable mild stress aimed at assessing depressive-like phenotypes, which revealed more significant trends in female mice regarding iNOS/Arg-1 and TNF-α/IL-10 compared with male mice (Liu et al., 2019), suggesting an increased pro-inflammatory tendency in females following exposure to stress. Nevertheless, conflicting findings have been reported about inflammatory dysregulation in depression across sexes. Despite women reporting more somatic symptoms and higher vulnerability to inflammation’s effects than men did, the connection between stress-related depressive conditions and low-grade inflammation exhibited more consistent prevalence in men than in women (Bekhbat and Neigh, 2018), and interestingly, C-reactive protein, a depression-related inflammatory biomarker, is exclusively associated with depression in men, not women (Bekhbat and Neigh, 2018). Additionally, neuroinflammation can result from neonatal immune activation (NIA) (Khantakova et al., 2022), and one study investigated whether depression-like behavior emerges following NIA in rodent models, which indicated that adult males with a history of NIA demonstrate pronounced depression-like behavior in response to aversive testing conditions, but not observed in females (Khantakova et al., 2022).

##### 3.2.1.4 Sex differences in depression’ s genetics

Genetic factors significantly impact depressive symptoms (Oliva et al., 2023), and multiple studies have unveiled sex-specific differences in the genetic underpinnings of depression (Labonté et al., 2017; Gururajan et al., 2019; Issler et al., 2020; Miyata et al., 2020; Paden et al., 2020), which this genetic divergence could enhance our understanding of the elevated prevalence of depression in females. To be specific, one study showed the presence of the neuron enrichment gene *LINC00473*, which has been identified as a sex-specific factor that contributes to stress resilience, and it was exclusively downregulated in the prefrontal cortex of depressed women, not men (Issler et al., 2020). Another study reported that downregulation of the *Dusp6* gene increased susceptibility to stress by enhancing the excitability of glutamatergic pyramidal neurons in the ventromedial prefrontal cortex through extracellular-signal-regulated kinase (ERK) signaling activation, especially in females rather than males, suggesting that the *Dusp6*-dependent enhancement of ERK signaling in the ventromedial prefrontal cortex leads to depressive symptoms in women, while a similar enhancement in men does not result in a noticeable effect (Labonté et al., 2017; Gururajan et al., 2019). Additionally, there exist a multitude of significant genes that vary between sexes and are linked to depression, like *ORM1*, *ORM2*, *RNF32*, *SLC25A5, Thbs1*, and *Cadps2* etc., manifesting sex-specific impacts on depression risk (Miyata et al., 2020; Paden et al., 2020). In conclusion, depression in both sexes may stem from genetic alterations, providing a foundation for comprehending the mechanisms behind depression.

#### 3.2.2 Sex differences in depression’s therapeutic opportunities

Current antidepressant treatments demonstrate low remission rates, with efficacy typically taking weeks to months after treatment initiation, and even after achieving treatment response, depressed patients show high rates of relapse to depressive disorders (Duman et al., 2019), which may be attributed, at least in part, to the complexities arising from sex differences in depression therapy. Table 2 shows current treatment strategies regarding sex differences in depression.

**Table 2:** Sex differences in depression’s therapeutic opportunities.

Drugs	Therapy effects	
	Male	Female
TCA	++	+
SSRI	+	++
ketamine	a	b
*HT*		
Estrogen	NA	++
Testosterone	++	NA
*Other therapies*		
Lifestyle interventions	+	++
vitamin D	NA	++
ECT	+	+

a The therapeutic efficacy is sensitive; b The therapeutic efficacy is lasting effects; + represents the general therapeutic efficacy; ++ represents the better therapeutic efficacy; − represents poor therapeutic efficacy; NA represents that the therapeutic efficacy is unclear.

AD, Alzheimer’s disease; ECT, Electroconvulsive therapy; HT, Hormone therapy; SSRIs, Selective serotonin reuptake inhibitors; TCA, Tricyclic.

##### 3.2.2.1 Antidepressants

Common antidepressants, such as tricyclics (TCAs) and selective serotonin reuptake inhibitors (SSRIs), are used for depression treatment, but sex-based disparities influence their effectiveness and side effects (Pavlidi et al., 2021). In humans, SSRIs are more effective in women, particularly among premenopausal women, while TCAs are more effective in men (LeGates et al., 2019). In rodents, females show greater sensitivity to ketamine, a fast-acting antidepressant, yet males experience more lasting effects (Franceschelli et al., 2015; LeGates et al., 2019; DuMont et al., 2023), hinting the presence of sex differences in the durability of antidepressant medications. Furthermore, sex differences were observed in the side effects of antidepressants, such as female patients displayed reduced tolerability of TCAs and experienced side effects including nausea, abnormal vision, somnolence, dizziness, and constipation, whereas male patients reported urinary complaints and experienced more significant sexual dysfunction (LeGates et al., 2019).

##### 3.2.2.2 Sex hormones

Sex hormones not only contribute to the pathogenesis of depression but also serve as one of essential approach in its treatment, and to be specific, both estrogens in women and androgens in men have been implicated in the treatment of depression. Estradiol therapies have demonstrated effectiveness in alleviating depressive symptoms, especially in women experiencing significant fluctuations or reductions in ovarian hormones, such as during the perimenopause and postpartum periods (Zhang J. et al., 2023). Likewise, testosterone therapies have shown efficacy in alleviating depression in men with hypogonadism (Eid et al., 2019). Additionally, sex hormones may influence the efficacy of antidepressants like SSRIs, and these drugs have been found to be more effective in premenopausal women compared with postmenopausal women. However, once HT is administered, the efficacy of SSRIs increases in postmenopausal women (Eid et al., 2019; Pavlidi et al., 2021).

Additionally, it is noteworthy that research has indicated that estrogens can enhance the antidepressant-like action of fluoxetine, a selective serotonin reuptake inhibitor, as well as desipramine and venlafaxine, which are selective noradrenaline reuptake inhibitors and mixed serotonin/noradrenaline reuptake inhibitors, respectively, substantially shortening the latency of their effects (Estrada-Camarena et al., 2010). Moreover, testosterone replacement therapy has yielded interesting results in the efficacy of antidepressants and an animal study assessing the impact of castration and testosterone replacement (1 mg per 100 g body weight) has shown that the effectiveness of fluoxetine is modulated by testosterone levels, such as the loss of the antidepressant effect of fluoxetine in castrated male rats (Busch et al., 2000).

##### 3.2.2.3 Other therapies

Besides common medications, adopting a healthy lifestyle can help alleviate depression in adults (Wang W. C. et al., 2022). Sex-specific differences in lifestyle interventions exist, with physical activity showing promise in improving women’s overall mental health than men (Zheng et al., 2023). Because low vitamin D levels are linked to higher depression risk, particularly in women, preventing vitamin D deficiency is crucial in mitigating their depression (Hinata et al., 2023). Electroconvulsive therapy (ECT) is the most effective treatment for MDD, demonstrating equal effectiveness regardless of sex (Blanken et al., 2023), signifying that remission rates following ECT remain unaffected by sex.

## 4 Sex differences in the relationship between AD and depression

AD represents the most prevalent form of dementia, and nearly all patients experience neuropsychiatric symptoms, with depression being one of the most common psychiatric disorders, in conjunction with cognitive and memory deficits (Lyketsos et al., 2011; Santos et al., 2016; Depp et al., 2023; Rodriguez Salgado et al., 2023; Tremblay et al., 2023). Importantly, depression not only manifests as an early symptom of AD dementia but also increases the risk of AD, and conversely, depressive disorders can also arise in response to cognitive decline due to AD (Elser et al., 2023; Liao et al., 2023), suggesting a crucial and bidirectional association between AD and depression. Significantly, different stages of depression are associated with the risk of dementia. Specifically, early depression has been consistently linked to more than doubling the risk of dementia, but research on late-life depression and the risk of dementia has been conflicting (Byers and Yaffe, 2011). Nevertheless, one study has suggested that late-life depression may increase the risk of AD (Diniz et al., 2013). Notably, both depression and AD display a significant sex disparity, with a higher prevalence observed among women (Wang et al., 2022a), but existing research on sex differences in the association between AD and depression produces incongruent findings. A recent study noted men appearing to be more susceptible to developing depressive symptoms following the onset of AD (Tremblay et al., 2023), which may arise from overlapping symptoms between AD dementia and depression, as well as the absence of consensus criteria to diagnose AD-related depression. Likewise, another study revealed that men diagnosed with depression have a higher risk of developing AD (Elser et al., 2023), but men may be less inclined to seek healthcare compared with women, resulting in consistent underestimation of the prevalence of depression among men. Altogether, these findings underscore the importance of investigating sex differences in the underlying association between AD and depression.

### 4.1 Sex differences in AD and depression’s mechanisms and genetics

#### 4.1.1 Stress, sex hormones and receptors of sex hormones

Stress is increasingly recognized as a catalyst for depression onset as well as a causal factor in the occurrence and progression of AD pathology (Sotiropoulos et al., 2008). One study conducted amid COVID-19 confinement revealed that, when exposed to identical stressors, individuals with amyloid positivity were prone to experiencing more pronounced depressive symptoms (Akinci et al., 2022). This observation implies that AD pathology might augment the frequency and intensity of depression in reaction to stressors. Of note, corticotropin-releasing hormone (CRH) tightly regulates the hypothalamic–pituitary–adrenal (HPA) axis, serving as a critical mediator in the stress response, and upon the action of CRH, adrenocorticotropic hormone (ACTH) is released, subsequently leading to the release of corticosteroids, such as glucocorticoids (Swaab et al., 2005). Long-term exposure to glucocorticoids, the primary stress hormones, can detrimentally affect the brain (Du et al., 2023), thereby acting as a risk factor for both AD and depression. Cortisol, the most prominent glucocorticoid in human, was found at higher levels in women than in men (Swaab et al., 2005), which may partially account for the higher incidence of depressed women with AD than depressed men. Moreover, a close interaction between the HPA axis and the hypothalamic–pituitary–gonadal (HPG) axis has been well-established, that is, the activating roles of sex hormones on the HPA axis have been demonstrated (Barel et al., 2018), indicating that the stress system is influenced by fluctuating levels of sex hormones and in AD-related depression. Both sexes revealed increased HPA axis activity and reduced HPG axis activity, and women exhibited lower plasma levels of estrogen, while men experienced reduced testosterone levels (Swaab et al., 2005).

In addition, it is noteworthy that sex hormones may regulate the activity of HPA axis in both humans and rats through their respective receptors, such as the estrogen receptor α or β (ERα and ERβ) and androgen receptors (ARs), which act directly on the CRH gene promoter (Lu et al., 2015). Thus, numerous studies have delved into the role of sex hormones receptors in mediating sex disparities in the AD and depression (Ishunina et al., 2002; Zhao et al., 2015; Rybka et al., 2022; He et al., 2023). To be specific, one study revealed that genetic polymorphisms of ERβ have shown an association with cognitive impairment and an elevated risk of AD, particularly in women as compared with men (Zhao et al., 2015). Of note, the nucleus basalis of Meynert (NBM) and the vertical limb of the diagoenal band of Broca (VDB) are pivotal cholinergic nuclei within the human basal forebrain, a complex that is impacted in AD (Ishunina et al., 2002). One study indicated that cytoplasmic ARs exhibited a marked reduction in the NBM and the VDB specifically among AD women but not among AD men (Ishunina et al., 2002) and this finding implies women’s higher susceptibility to AD compared with those of men. Significantly, androgen actions through ARs reduce stress-related behaviors and HPA axis responses, resulting in an amelioration of depressive mood especially among men (Rybka et al., 2022). Moreover, ERβ expression is enriched in the dorsal raphe nucleus (DRN), a region closely linked to emotion regulation (He et al., 2023). Deletion of DRN-specific ERβ leads to slightly elevated anxiety in women but does not affect anxiety levels in men (He et al., 2023), highlighting the sex-specific involvement of DRN ERβ in emotional behavior regulation.

#### 4.1.2 Hippocampal formation

The hippocampus, a crucial structure for memory and cognition, holds a key role in depression and AD development (Idunkova et al., 2023; Ortega-Cruz et al., 2023). Sex differences in hippocampal volume (HV) may contribute to depression and AD pathogenesis, such as women may experience faster hippocampal atrophy, resulting in a greater decrease in hippocampal volume than men (DuMont et al., 2023), and thus, women could be more susceptible to experiencing greater cognitive decline in the context of depression in AD compared with men. Decreased hippocampal volume is associated with increased AD pathology, which is more pronounced in women, especially in the MCI stage (Yagi and Galea, 2019). However, while both depressed men and women demonstrated a more pronounced decline in hippocampal volume, the greater decline was observed in men compared with women (Yagi and Galea, 2019). In rats with AD, higher hippocampal tumor necrosis factor (TNF)-α levels induced an upsurge in depressive symptoms in both sexes. Intriguingly, female AD rats showed fewer depression-related behaviors, linked to increased hippocampal brain-derived neurotrophic factor (BDNF), crucial for neuronal differentiation and preservation, suggesting that BDNF mitigates AD cognitive impairment and holds antidepressant properties, observed in females, but not in males with AD (Linnemann and Lang, 2020; Naghibi et al., 2021). Moreover, the combination of estrogens with antidepressants like fluoxetine has been noted to enhance neurogenesis and dendritic arborization in the hippocampus, correlating with more significant antidepressant-like effects in female rats (Vega-Rivera et al., 2015).

#### 4.1.3 Neuroinflammation

Neuroinflammation has a significant impact on the progression of both AD and depression (Linnemann and Lang, 2020). Elevated levels of TNF-α and interleukin-6 (IL-6) within the peripheral blood are closely associated with MDD (Dowlati et al., 2010). Likewise, heightened levels of IL-1β, IL-6, IL-12, IL-18, TGF-β, and TNF-α are linked to AD (Swardfager et al., 2010), and importantly, patients diagnosed with both AD and depression demonstrate the highest levels of IL-6 and TNF-α in their circulatory system (Santos et al., 2016). Interestingly, sex differences emerge in the neuroinflammatory mechanisms underlying AD and depression, that is, women with AD and depression appear more susceptible to the impact of neuroinflammation compared with men (Bekhbat and Neigh, 2018; Cui et al., 2023). Remarkably, the sex differences in the function of microglia may be one of the contributing factors to the variations in the prognosis and susceptibility of depression and AD between men and women (Chen et al., 2021) and in several study, female mice with both diseases displayed a marked rise in counts of microglia compared with male mice (Bekhbat and Neigh, 2018; O'Neill et al., 2022). In addition, one study examined the gene expression profiles of microglia isolated from the hippocampus and cortex of female and male *AppNL-G-F* mice and found that microglia in female mice progressed faster on the Activated response microglia (ARMs) trajectory compared with those of male mice (Sala Frigerio et al., 2019). Similarly, another study demonstrated that microglia in healthy women play a protective role, yet in the presence of AD, the protective effect of female microglia diminishes, instead contributing to the disease progression of AD (Chen et al., 2021). Nevertheless, one recent study suggested that chronic stress significantly modifies the morphology and the behavior of microglia in a sex-specific manner, and to be specific, males were more susceptible to stress, displaying depression-like behaviors and microglial hypertrophy, whereas females showed microglial remodeling in the NAc (atrophy), and did not exhibit depression-like behaviors (Gaspar et al., 2022). Overall, these findings pinpoint the importance of exploring the specific role of sex differences with microglia in the association between AD and depression. However, the underlying mechanisms remain equivocal, necessitating further exploration in future studies.

#### 4.1.4 Sex differences in AD and depression’s genetics

Both AD and depression are not only affected by genetic factors, but also intriguing sex differences have been observed in the genetic susceptibility (Labonté et al., 2017; Nebel et al., 2018), which hold promise in elucidating pathogenesis and enhancing early detection as well as management of AD coexisting with depression. Herein, we discuss several sex-specific genetic factors in AD and depression.

##### 4.1.4.1 Sex-specific genetics

One genome-wide association study involving over 12,000 individuals with AD was the first to employ single-nucleotide polymorphisms for quantifying the heritability of psychosis within AD. The study uncovered a favorable genetic correlation between depressive symptoms and psychosis of AD (DeMichele-Sweet et al., 2021). Furthermore, the first multi-omics investigation on a genome-wide scale delved into the role of epigenome alterations and gene expression in the risk of comorbid depression in patients with late-onset AD and elucidated sex-specific disparities in the expression of gene that contribute to the manifestation of depressive symptoms in late-onset AD (Upadhya et al., 2022). Specifically, there were 25 differentially expressed genes associated with depression in AD men, and *CHI3L2*, involved in multiple inflammatory reactions of depression in late-onset AD, was the most upregulated gene, while only three differentially expressed genes were associated with depression in AD women (Upadhya et al., 2022).

##### 4.1.4.2 TREM2 and APOE

Microglial activation plays an integral role in neuroinflammation (Santos et al., 2016) and constitutes a well-established pathological hallmark in the brains of AD patients (Long and Holtzman, 2019) while exerting a significant influence on the regulation of depressive disorders (Jia et al., 2021). Additionally, the *TREM2* gene is known to play a significant role in microglial activation and survival, as well as it has been recognized as a risk factor for depression in AD (Wang et al., 2022a; Wu et al., 2023). Likewise, the *ApoE-ε4* allele influences the microglial responses and has been confirmed as a risk factor for both AD and depression (Santos et al., 2016; Long and Holtzman, 2019). Of note, minimal depressive symptoms (MDSs) were discovered to correlate not only with elevated cerebrospinal fluid (CSF) amyloid markers but also with an 83% heightened likelihood of developing AD in elderly adults without dementia (Xu et al., 2021). One study demonstrated a strong relationship between CSF-soluble TREM2 (sTREM2) and MDSs but no significant sex differences. However, the researchers found that women with MDSs may have stronger associations with increased amyloid burdens and impaired function of microglia than men do (Wang et al., 2022a). Furthermore, one study revealed that early depression is associated with amyloid pathology mediated by microglial activation, especially in the absence of ApoE-ε4 (Wang et al., 2022a). However, another study demonstrated that combing ApoE-ε4 can significantly improve the predictive accuracy of depressive symptoms for predicting conversion from MCI to AD dementia, specifically in women (Kim et al., 2015). Overall, it is crucial to conduct sex-stratified genetics studies in depression and AD. Even if no significant sex differences are found, as they significantly contribute to the progress of precision medicine.

### 4.2 Sex differences in depression and AD’s therapeutic opportunities

#### 4.2.1 Sex hormones therapy

Sex hormone replacement therapy (HRT) effectively alleviates depressive symptoms in AD patients (Swaab et al., 2005) (Table 3). One study links sex hormone level change to sex differences in AD patients with depression (DuMont et al., 2023). Premature estrogen decline heightens susceptibility of women to AD and depression compared with men (Sochocka et al., 2023), suggesting women could benefit more from HRT. However, despite estrogen therapy studies mostly focusing on females, an increasing acknowledgment of the significant contributions of estrogens to male brain has emerged (Gillies and McArthur, 2010). As a result, HRT presents a promising avenue for mitigating depressive symptoms and AD-associated manifestations in both men and women. However, it is essential to recognize that HRT is not without its constraints, i.e., estrogen therapy could potentially elevate a woman’s vulnerability to ovarian, endometrial, and breast cancers (Crespo-Castrillo and Arevalo, 2020). Additionally, the utilization of androgen deprivation therapy (ADT) might be linked to an exacerbated risk of experiencing depression and cognitive impairment (Siebert et al., 2020).

**Table 3:** Sex differences in depression and AD’s therapeutic opportunities.

Drugs		Therapy effects	
		Male	Female
Sex hormones	HRT	+	++
Neuroinflammation	IDO	++	+

+ represents the general therapeutic efficacy; ++ represents the better therapeutic efficacy.

AD, Alzheimer’s disease; IDO, indoleamine-2,3-dioxygenase; HRT, Sex hormone replacement therapy.

#### 4.2.2 The treatment of neuroinflammation

The significant role of neuroinflammation in the pathogenesis of AD and depression is widely acknowledged (Santos et al., 2016). Elevated levels of pro-inflammatory cytokines stimulate the overexpression of indoleamine-2,3-dioxygenase (IDO), an enzyme that converts tryptophan into kynurenine primarily within microglia. This enzymatic conversion leads to reduced tryptophan availability due to increased kynurenine production, potentially obstructing the synthesis of serotonin, which may contribute to depression among AD patients (Santos et al., 2016). Thus, inhibiting IDO or kynurenine hydroxylase to target the kynurenine pathway is a promising approach for treating both MDD and AD (Table 3). Significantly, animal experiments have provided evidence to support this strategy (O'Connor et al., 2009; Yu et al., 2015), and to be specific, emerging research indicates that there may be sex differences in the effectiveness of this treatment. For instance, administering the novel IDO inhibitor DWG-1036 to 3xTg-AD mice aged 2 to 6 months resulted in enhanced cognitive function and reduced depression-related behaviors in AD. Furthermore, the treatment’s impact on depression in AD was observed to be more pronounced in male mice than in female mice (Fertan et al., 2019).

#### 4.2.3 Transcranial magnetic stimulation (rTMS)

Transcranial magnetic stimulation (rTMS), as a safe and non-invasive brain neuromodulation method, has demonstrated effectiveness in treating AD and depression (Xu et al., 2022; Zhang et al., 2022). Interestingly, previous research has indicated potential sex differences in cortical plasticity induced by rTMS. Specifically, a study revealed ovarian hormone-dependent excitability in the primary motor cortex (M1) in females, which is absent in males. Women exhibited higher rTMS-induced motor evoked potentials (MEP) during the late menstrual cycle (Zeng et al., 2020). However, no sex differences were found in TMS-induced cortical excitability between men and women in young adulthood. In contrast, internal age-related differences in cortical excitability within each sex showed more pronounced decreases in excitability in older women compared to men (Zeng et al., 2020). These findings suggest the possibility of sex-dependent rTMS treatment for AD and depression. Notably, a study comparing chronic rTMS effects on forced swim behaviors in male and female rats, to validate rTMS’s antidepressant effect, indicated that female rats consistently showed higher activity levels in the Forced Swim Test (FST) (Yang et al., 2007). Another study indicated that women, especially during periods of high estradiol, appear particularly sensitive to rTMS treatment, with a 1.37 times higher probability of major depressive disorder remission in women compared to men (Hanlon and McCalley, 2022). Further research demonstrated more marked improvements in depression in women following rTMS treatment, with no significant changes noted in men (Desai et al., 2023). However, direct research exploring sex differences in rTMS treatment for AD is currently limited, with only one study suggesting that TMS may have a more pronounced impact on cognitive performance in women than in men (Zeng et al., 2020).

## 5 Conclusion and future directions

Accumulating evidence supports the significant influence of sex on depression and AD. Specifically, women experience higher prevalence of both conditions than men do, possibly leading to differences in treatment outcome (Hampel et al., 2018; Wang et al., 2022a; Cui et al., 2023; Tseng et al., 2023), suggesting the imperative of crafting personalized approaches based on sex differences. Sex disparities unveil crucial biological mechanisms underlying AD and depression etiology and progression, encompassing neuroinflammation, hormones, brain structure, and more. There is mounting evidence indicating sex-specific susceptibility to the effects of the ApoE-ε4 allele and sex-specific genes. Additionally, interactions between sex and treatment responses targeting hormones, neuroinflammation therapy, etc., have been observed. Taken together, evidence confirms sex as a vital contributor to phenotypic variability in AD and depression, warranting consideration in clinical practice and preclinical research. Substantial enhancements are necessary for the analysis and reporting of sex differences in both clinical and preclinical studies to produce robust evidence capable of guiding changes in clinical practice.

Emerging research has illuminated the influence of sex on AD and depression, offering promising avenues for therapeutic advancement. Recent investigations highlight the significant amelioration of cognitive impairment and depression in AD patients through the administration of minocycline, a well-established anti-inflammatory drug (Cheng et al., 2023). Zinc (Zn^2+^), a vital trace element associated with CNS neuroinflammation (Liu et al., 2023), has been identified as a therapeutic target for depression in AD through brain Zn^2+^ homeostasis maintenance (Wang B. et al., 2023). However, these studies have overlooked the impact of sex differences, highlighting the imperative for future research in this domain. Furthermore, while beneficial gut bacteria like *Lactobacillus* and *Bifidobacterium* alleviate depression in AD via gut microbiota modulation (Kerry et al., 2018; Holingue et al., 2020), the sexual differences of the gut microbiome in AD and depression remain underexplored. This gap warrants exploration of sex-based gut microbiome treatments for a tailored precision medicine approach. Additionally, nonpharmacological methods such as transcranial pulse stimulation, aromatherapy, psychosocial interventions, social interventions, and music interventions, among others (Dhippayom et al., 2022; Bavarsad et al., 2023; Chen X. et al., 2023; McAleer et al., 2023; Tan et al., 2023; Vuijk et al., 2023), hold significant promise as strategies to enhance the management of depression and AD. Moving forward, the development of a precision medicine framework, integrating knowledge gained from sex-related distinctions, could transform the future management of AD and depression, ultimately contributing to substantial improvements in the quality of life for almost all patients.

The study of sex differences in depression in AD faces several challenges that warrant attention in future research. For example, although health agencies require comparable representation of female and male subjects in preclinical and clinical trials, a significant imbalance in the utilization of male and female subjects still persists in many studies, and most basic research also has heavily relied on male rodent cells or animals as disease models, disregarding investigations involving both sexes (Pallier et al., 2022; DuMont et al., 2023). Notably, despite the remarkable technological advancements made in neuroscience in the 21st century, the conceptual progress in investigating diseases across sexes, particularly in comprehending and effectively treating men and women, has fallen behind (Bangasser and Cuarenta, 2021). Moving forward, if studies pay more attention to the differences brought about by sex in etiology or genetics, perhaps more effective and universally applicable treatments for AD and depression can be developed in the near future. We believe that the research field of sex differences in depression and AD holds tremendous promise and is anticipated to flourish in the future.

## Author contributions

Y-HC: Conceptualization, Methodology, Writing – original draft, Writing – review & editing. Z-BW: Supervision, Writing – review & editing. X-PL: Writing – review & editing. J-PX: Writing – review & editing. Z-QM: Conceptualization, Funding acquisition, Methodology, Writing – review & editing.
